# Crystal structures of 6-nitro­quinazolin-4(3*H*)-one, 6-amino­quinazolin-4(3*H*)-one and 4-amino­quinazoline hemi­hydro­chloride dihydrate

**DOI:** 10.1107/S2056989021008823

**Published:** 2021-09-03

**Authors:** Kambarali Turgunov, Mirjalol Ziyadullaev, Farkhod Khoshimov, Rikhsiboy Karimov, Burkhon Elmuradov

**Affiliations:** a S. Yunusov Institute of the Chemistry of Plant Substances, Academy of Sciences of Uzbekistan, Mirzo Ulugbek Str., 77, Tashkent 100170, Uzbekistan; b Turin Polytechnic University in Tashkent, Kichik Khalka Yuli Str. 17, Tashkent 100095, Uzbekistan; c Namangan Institute of Engineering and Technology, Kosonsoy Str., 7, Namangan 160115, Uzbekistan

**Keywords:** crystal structure, 4-amino­quinazoline, 6-amino­quinazolin-4(3*H*)-one, 6-nitro­quinazolin-4(3*H*)-one, hemi­hydro­chloride

## Abstract

The title compounds, 6-nitro­quinazolin-4(3*H*)-one (C_8_H_5_N_3_O_3_; **I**), 6-amino­quinazolin-4(3*H*)-one (C_8_H_7_N_3_O; **II**) and 4-amino­quinazolin-1-ium chloride–4-amino­quinazoline–water (1/1/2), (C_8_H_8_N_3_^+^·Cl^−^·C_8_H_7_N_3_·2H_2_O; **III**) were synthesized and their structures were determined by single-crystal X-ray analysis. In the crystals of **I** and **II**, the quinazoline mol­ecules form hydrogen-bonded dimers *via* N—H⋯O inter­actions. The dimers are connected by weak inter­molecular C—H⋯N and C—H⋯O hydrogen bonds, forming a layered structure in the case of **I**. In the crystal of **II**, N—H⋯N and C—H⋯O inter­actions link the dimers into a three-dimensional network structure. The asymmetric unit of **III** consists of two quinazoline mol­ecules, one of which is protonated, a chloride ion, and two water mol­ecules. The chloride anion and the water mol­ecules form hydrogen-bonded chains consisting of fused five-membered rings. The protonated and unprotonated quinazolin mol­ecules are linked to the chloride ions and water mol­ecules of the chain by their amino groups.

## Abstract 2

6-Nitro­quinazolin-4(3*H*)-one (C_8_H_5_N_3_O_3_), 6-amino­quinazolin-4(3*H*)-one (C_8_H_7_N_3_O) and 4-amino­quinazoline hemi­hydro­chloride dihydrate (C_16_H_19_N_6_O_2_) were synthesized and their structures were determined by single-crystal X-ray analysis.

## Chemical context   

Heterocyclic compounds play an important role in the lives of plant and living organisms because of their properties, including anti-inflammatory (Azab *et al.*, 2016 ▸), anti­tumor (Ishikawa *et al.*, 2009 ▸), anti­viral (De Clercq & Field, 2006 ▸) and other activities (Ding *et al.*, 1999 ▸). Quinazoline derivatives occupy a distinct position among nitro­gen-containing heterocycles because of their wide spectrum of pharmaceutical and biopharmaceutical properties, amongst them anti­cancer (Chandregowda *et al.*, 2009 ▸), anti­bacterial (Anti­penko *et al.*, 2009 ▸), anti-inflammatory (Alagarsamy *et al.*, 2007 ▸), anti­tuberculosis (Nandy *et al.*, 2006 ▸), anti­hypertension (Hess *et al.*, 1968 ▸) and anti­diabetic (Paneersalvam *et al.*, 2010 ▸) activities.
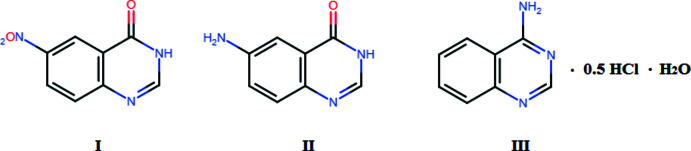


In line with this, we synthesized 6-nitro­quinazolin-4(3*H*)-one (**I**), 6-amino­quinazolin-4(3*H*)-one (**II**) and 4-amino­quinazoline hemi­hydro­chloride dihydrate (**III**), which are important inter­mediates in drug synthesis, and their crystal structures were determined. The hemi-protonated structures may be useful for the preparation of materials important to various branches of science, ranging from biology to nanodevice fabrication and to pharmaceuticals (Perumalla *et al.*, 2013 ▸).

## Structural commentary   

**Figure 1:**
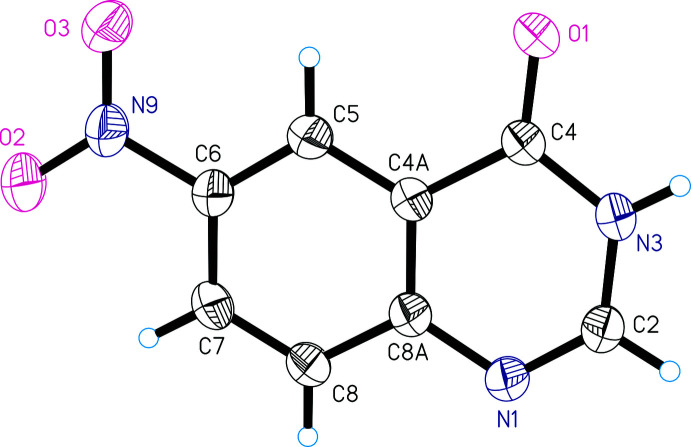
The mol­ecular structure of 6-nitro­quinazolin-4(3*H*)-one (**I**), with displacement ellipsoids drawn at the 50% probability level.

Compound **I** crystallizes in the triclinic space group *P* 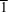 with one mol­ecule in the asymmetric unit. As a whole, the mol­ecule is nearly planar. The nitro group is rotated slightly with respect to the quinazoline ring system, the C5—C6—N9—O3 and C7—C6—N9—O2 torsion angles being 6.0 (3) and 4.9 (4)°, respectively. All bond lengths and angles are normal and in good agreement with those reported previously (Liao *et al.*, 2018 ▸; Yong *et al.*, 2008 ▸). Fig. 1 ▸ shows the mol­ecular structure of **I** in the solid state. Selected geometric parameters are listed in Table 1 ▸.

**Figure 2:**
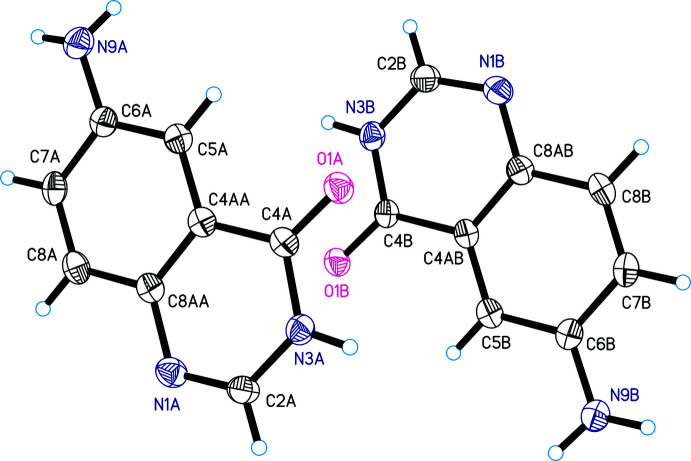
The mol­ecular structure of 6-amino­quinazolin-4(3*H*)-one (**II**), showing the two independent mol­ecules, with displacement ellipsoids drawn at the 50% probability level.

Compound **II** crystallizes in the ortho­rhom­bic space group *Pca*2_1_ with two crystallographically independent mol­ecules, *A* and *B*, in the asymmetric unit (Fig. 2 ▸). All the atoms of the mol­ecule (except the amino-group hydrogens) lie in the same plane. The nitro­gen atom of the amino group is somewhere between the *sp^2^* and *sp^3^* hybridized states, the sum of the valence angles at the nitrogen atom being 349 and 342° in mol­ecules *A* and *B*, respectively. All bond lengths and angles are normal. Selected geometric parameters are listed in Table 2 ▸.

**Figure 3:**
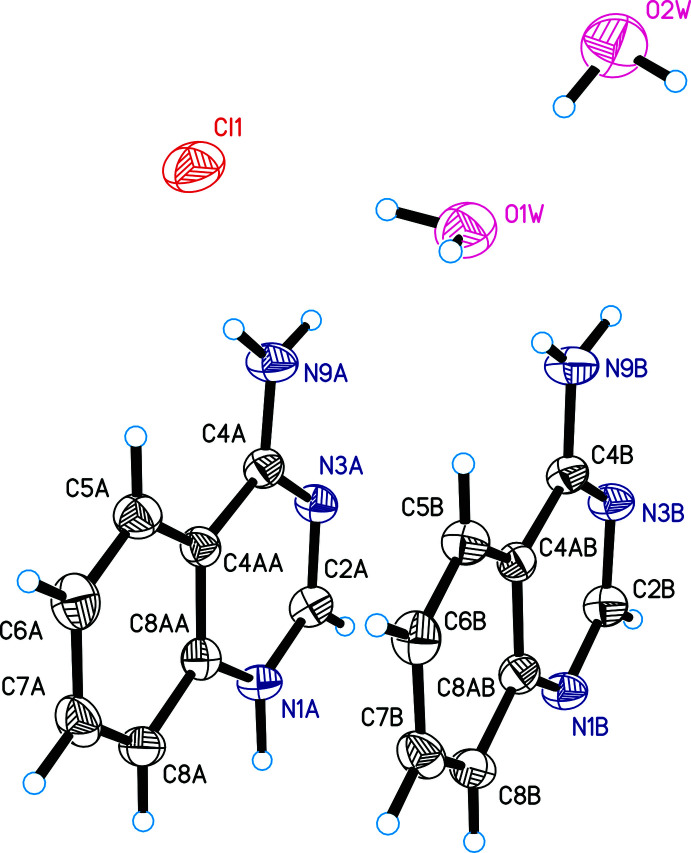
The asymmetric unit of compound **III** with displacement ellipsoids drawn at the 50% probability level.

In the case of compound **III**, there are protonated (*A*) and unprotonated (*B*) 4-amino­quinazoline mol­ecules (Fig. 3 ▸) in the asymmetric unit and they both have a planar structure. Mol­ecule *A* is protonated at the N1 nitro­gen atom and this leads to an elongation of the N1—C2 and N3—C4 bonds and a shortening of the C2—N3 and C4—N9 bonds with respect to the unprotonated mol­ecule *B*. In both *A* and *B*, the nitro­gen atom of the amino group is in an *sp^2^* hybridized state. The sum of the valence angles around the nitro­gen atoms in mol­ecules *A* and *B* are 360 and 359°, respectively, and the carbon-to-amino group nitro­gen bond lengths C4—N9 are shorter than the bond lengths observed in compound **II** (Table 3 ▸).

## Supra­molecular features   

**Figure 4:**
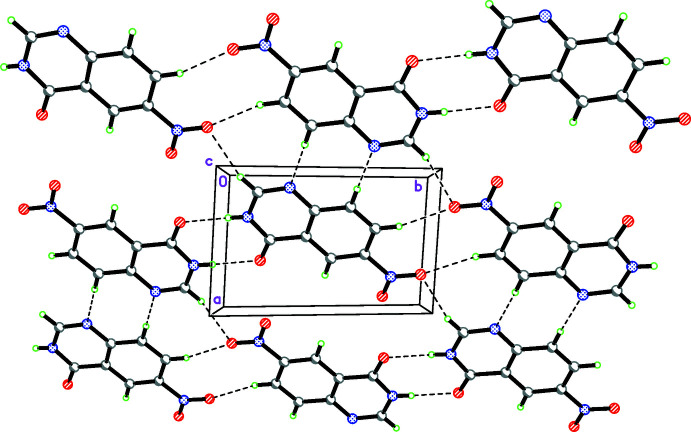
Hydrogen bonding in the crystal of 6-nitro­quinazolin-4(3*H*)-one (**I**). Colour code: C grey, H green, N blue, O red.

In the crystal of **I**, inter­molecular N—H⋯O hydrogen bonds link the mol­ecules into centrosymmetric dimers, forming 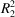
(8) motifs. Other head-to-head 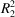
(10) and 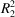
(8) motifs are formed by weak inter­molecular C—H⋯O and C—H⋯N hydrogen bonds, producing layers parallel to the (1
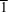
2) plane (Table 4 ▸, Fig. 4 ▸). In addition, an 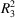
(8) ring motif is formed by the inter­actions between three adjacent mol­ecules. The layers are linked though π–π stacking inter­actions with centroid–centroid distances of 3.8264 (13) and 3.9600 (14) Å into a three-dimensional network.

**Figure 5:**
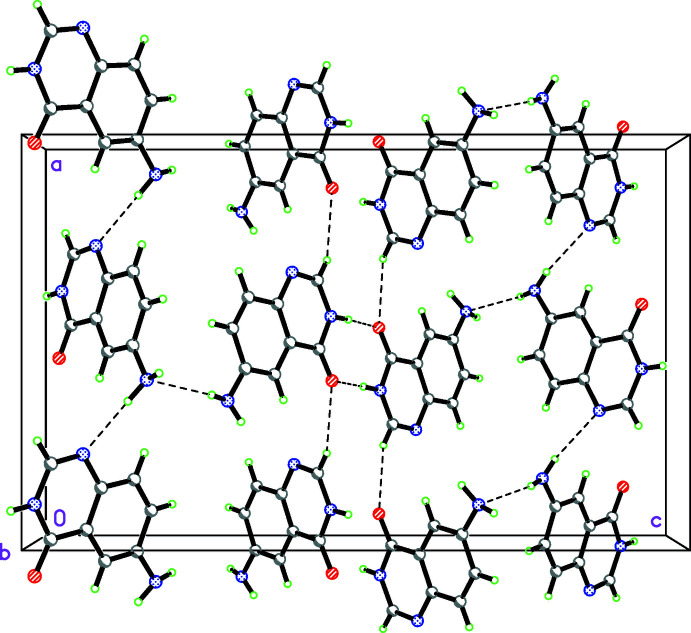
Hydrogen bonding in the crystal of 6-amino­quinazolin-4(3*H*)-one (**II**). Colour code: C grey, H green, N blue, O red.

The two independent mol­ecules of compound **II** are related by a pseudo-center of symmetry and are linked by two N—H⋯O hydrogen bonds, forming an 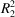
(8) motif. An N—H⋯N hydrogen bond generates a three-dimensional network (Table 5 ▸, Fig. 5 ▸).

**Figure 6:**
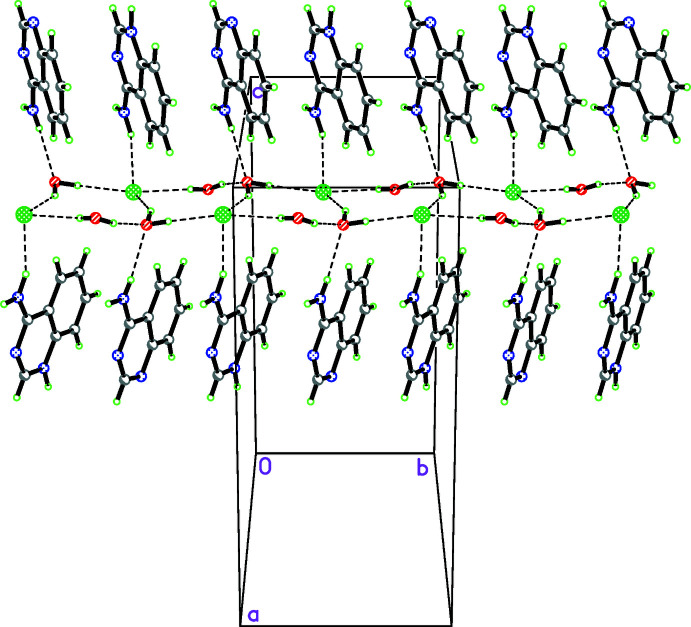
Part of the crystal structure of **III** showing the hydrogen-bonding scheme. Colour code: C grey, H light green, Cl bright green, N blue, O red.

The packing analysis of **III** shows that the protonated and unprotonated 4-amino­quinazoline mol­ecules are linked by inter­molecular N—H⋯N hydrogen bonds, forming pseudo-centrosymmetric dimers characterized by a donor–acceptor distance of 2.786 (3) Å. Other N—H⋯N hydrogen bonds form centrosymmetric 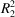
(8) ring motifs. The chloride anion and water mol­ecules form hydrogen-bonded chains consisting of fused five-membered rings with the participation of two chloride anions and three water mol­ecules. A chain of rings runs through the twofold screw axis parallel to the [010] direction (Fig. 6 ▸). The protonated and unprotonated quinazoline mol­ecules link to the chain *via* N—H⋯Cl and N—H⋯Ow hydrogen bonds from the lower and upper side (Table 6 ▸, Fig. 6 ▸). The chain direction corresponds to the smallest unit-cell edge and such *self-assembly* of mol­ecules has also been observed in other quinazoline hydro­chloride crystals (Tashkhodzhaev *et al.*, 1995 ▸; Turgunov *et al.*, 1998 ▸, 2003 ▸). The above mentioned N—H⋯N hydrogen bonds link the mol­ecules into a three-dimensional network. The crystal structure of **III** is stabilized by π–π inter­actions [centroid–centroid distances in the range 3.4113 (16)–3.9080 (18) Å].

## Database survey   

A search of the Cambridge Structural Database (CSD, version 5.41, including the update of January 2020; Groom *et al.*, 2016 ▸) confirmed that three related compounds had been structurally characterized in which the benzene ring of the quinazolin-4(3*H*)-ones contains a nitro group [refcodes GAPPUK (Yu *et al.*, 2012 ▸), GISXOW (Yong *et al.*, 2008 ▸) and RUGKEK (Wu *et al.*, 2009 ▸)].

The crystal structures of quinazolin-4(3*H*)-one and its first metal coordination compound have also been reported [BIHJIO (Liao *et al.*, 2018 ▸) and NALFEN (Turgunov & Englert, 2010 ▸)].

## Synthesis and crystallization   

**Compound I:** In a three-necked flask equipped with a mechanical stirrer and reflux condenser, quinazolin-4(3*H*)-one (22.5 g, 0.15 mol) was dissolved in 78 ml of concentrated sulfuric acid at 303 K for 1 h. Then a nitrating mixture (21 ml of nitric acid and 18 ml of concentrated sulfuric acid) was added to the flask under vigorous stirring of the mixture. The reaction mixture was stirred for another hour, maintaining a temperature not higher than 303 K, and then for another hour at room temperature. At room temperature, 45 ml of nitric acid were added dropwise to the reaction mixture over a period of 1 h. The reaction mixture was left at room temperature for 10 h. The contents of the flask were poured into a dish containing ice, the resulting precipitate was filtered off, washed with water and dried and recrystallized from ethanol to obtain 25.7 g of pure compound **I** as single crystals in 87.4% yield, m.p. 560–562 K.

**Compound II:** In a three-necked flask equipped with a mechanical stirrer and reflux condenser, 12.6 g (56 mmol) of tin (II) chloride dihydrate (SnCl_2_·2H_2_O) were cooled in an ice bath and 16.98 ml of concentrated (36%) HCl were added, then 3 g (16 mmol) of quinazolin-4-one as a suspension in 20 ml of ethanol and 7 ml of HCl (36%) were added portionwise with stirring of the mixture. The reaction was carried out for 10-15 minutes at ∼273 K, 30 min at room temperature and 2 h in a water bath (∼363 K). The reaction mixture was left overnight at room temperature, diluted with water, and brought to a strongly alkaline medium (pH = 10–11) with 10% of sodium hydroxide, in which the expected 6-amino-3*N*-quinazoline-4-one was dissolved, so that the chloride was brought to a neutral medium in the presence of acid, and precipitated when converted to an alkaline medium with ammonia. The precipitate was filtered, washed with water until it reached a neutral medium, and dried at room temperature. The precipitate was recrystallized from ethanol and 6.67 g of pure compound **II** were obtained representing an 88.1% yield, m.p. 589–591 K.

**Compound III:** Crystals of compound **III** were obtained as a minor additional product in the reaction of 4-chloro­quinazoline with ammonia.

## Refinement   

Crystal data, data collection and structure refinement details are summarized in Table 7 ▸. C-bound H atoms were placed in calculated positions and refined to ride on their parent atoms: C—H = 0.93 Å with *U*_iso_(H) = 1.2*U*_eq_(C). Hydrogen atoms of the water mol­ecules and those bonded to nitro­gen atoms were located in electron density difference maps and were freely refined.

## Supplementary Material

Crystal structure: contains datablock(s) I, II, III, GLOBAL. DOI: 10.1107/S2056989021008823/dj2030sup1.cif

Structure factors: contains datablock(s) I. DOI: 10.1107/S2056989021008823/dj2030Isup2.hkl

Structure factors: contains datablock(s) II. DOI: 10.1107/S2056989021008823/dj2030IIsup3.hkl

Structure factors: contains datablock(s) III. DOI: 10.1107/S2056989021008823/dj2030IIIsup4.hkl

Supporting information file. DOI: 10.1107/S2056989021008823/dj2030Isup5.cml

Supporting information file. DOI: 10.1107/S2056989021008823/dj2030IIsup6.cml

Supporting information file. DOI: 10.1107/S2056989021008823/dj2030IIIsup7.cml

CCDC references: 2104939, 2104938, 2104937

Additional supporting information:  crystallographic information; 3D view; checkCIF report

## Figures and Tables

**Table 1:** Selected bond lengths (Å) for **I** [Chem scheme1]

N1—C2	1.287 (3)	N3—C4	1.366 (3)
C2—N3	1.354 (3)	C6—N9	1.464 (3)

**Table 2:** Selected bond lengths (Å) for **II** [Chem scheme1]

N1*A*—C2*A*	1.291 (5)	N1*B*—C2*B*	1.290 (5)
C2*A*—N3*A*	1.369 (4)	C2*B*—N3*B*	1.364 (4)
N3*A*—C4*A*	1.376 (4)	N3*B*—C4*B*	1.366 (4)
C6*A*—N9*A*	1.374 (4)	C6*B*—N9*B*	1.392 (5)

**Table 3:** Selected bond lengths (Å) for **III** [Chem scheme1]

N1*A*—C2*A*	1.315 (4)	N1*B*—C2*B*	1.309 (4)
C2*A*—N3*A*	1.328 (4)	C2*B*—N3*B*	1.340 (4)
N3*A*—C4*A*	1.363 (4)	N3*B*—C4*B*	1.347 (4)
C4*A*—N9*A*	1.293 (4)	C4*B*—N9*B*	1.323 (4)

**Table 4:** Hydrogen-bond geometry (Å, °) for **I** [Chem scheme1]

*D*—H⋯*A*	*D*—H	H⋯*A*	*D*⋯*A*	*D*—H⋯*A*
N3—H3⋯O1^i^	0.80 (3)	2.02 (3)	2.814 (2)	178 (4)
C8—H8⋯N1^ii^	0.93	2.53	3.450 (3)	172
C2—H2⋯O2^iii^	0.93	2.57	3.466 (4)	163
C7—H7⋯O2^iv^	0.93	2.56	3.437 (3)	158

Symmetry codes: (i) -x+1, -y, -z+1; (ii) -x, -y+1, -z+2; (iii) x-1, y-1, z; (iv) -x+1, -y+2, -z+2.

**Table 5:** Hydrogen-bond geometry (Å, °) for **II** [Chem scheme1]

*D*—H⋯*A*	*D*—H	H⋯*A*	*D*⋯*A*	*D*—H⋯*A*
N9*A*—H9*AA*⋯N9*B* ^i^	0.93 (4)	2.55 (4)	3.435 (5)	160 (4)
N9*A*—H9*AB*⋯N1*A* ^ii^	0.81 (4)	2.34 (4)	3.144 (5)	170 (4)
N9*B*—H9*BB*⋯N1*B* ^iii^	0.91 (4)	2.19 (4)	3.092 (5)	174 (4)
N3*A*—H3*A*⋯O1*B* ^iv^	0.95 (3)	1.89 (3)	2.832 (4)	175 (3)
N3*B*—H3*B*⋯O1*A* ^v^	0.92 (4)	1.93 (4)	2.847 (3)	173 (4)

Symmetry codes: (i) -x+1, -y+1, z-{\script{1\over 2}}; (ii) x-{\script{1\over 2}}, -y+1, z; (iii) x+{\script{1\over 2}}, -y, z; (iv) x, y-1, z; (v) x, y+1, z.

**Table 6:** Hydrogen-bond geometry (Å, °) for **III** [Chem scheme1]

*D*—H⋯*A*	*D*—H	H⋯*A*	*D*⋯*A*	*D*—H⋯*A*
N1*A*—H1*A*⋯N1*B* ^i^	1.03 (3)	1.76 (3)	2.786 (3)	173 (3)
N9*A*—H9*AA*⋯N3*B* ^ii^	0.91 (4)	2.00 (4)	2.907 (4)	175 (3)
N9*A*—H9*AB*⋯Cl1	0.94 (5)	2.34 (5)	3.206 (2)	153 (5)
N9*B*—H9*BA*⋯N3*A* ^ii^	0.96 (4)	2.12 (4)	3.074 (4)	174 (3)
N9*B*—H9*BB*⋯O1*W*	0.79 (4)	2.22 (3)	2.999 (4)	167 (4)
O1*W*—H1*W*1⋯Cl1	0.90 (3)	2.25 (3)	3.157 (4)	178 (6)
O1*W*—H2*W*1⋯Cl1^iii^	0.89 (4)	2.37 (4)	3.183 (3)	151 (7)
O2*W*—H1*W*2⋯O1*W*	0.91 (7)	1.96 (7)	2.857 (5)	169 (6)
O2*W*—H2*W*2⋯Cl1^iv^	0.89 (5)	2.40 (5)	3.215 (4)	153 (5)

Symmetry codes: (i) -x+{\script{1\over 2}}, y+{\script{1\over 2}}, -z+{\script{1\over 2}}; (ii) -x+1, -y+1, -z+1; (iii) -x+{\script{1\over 2}}, y-{\script{1\over 2}}, -z+{\script{3\over 2}}; (iv) x, y-1, z.

**Table 7:** Experimental details

	**I**	**II**	**III**
Crystal data			
Chemical formula	C_8_H_5_N_3_O_3_	C_8_H_7_N_3_O	C_8_H_8_N_3_^+^·Cl^−^·C_8_H_7_N_3_·2H_2_O
*M*_r_	191.15	161.17	362.82
Crystal system, space group	Triclinic, *P*\overline{1}	Orthorhombic, *P* *c* *a*2_1_	Monoclinic, *P*2_1_/*n*
Temperature (K)	293	293	298
*a*, *b*, *c* (Å)	5.5587 (9), 8.6673 (13), 8.7649 (12)	13.4535 (5), 4.9510 (2), 21.6188 (8)	14.3512 (12), 7.5867 (6), 16.2282 (9)
α, β, γ (°)	105.654 (12), 98.560 (13), 90.784 (13)	90, 90, 90	90, 93.544 (7), 90
*V* (Å^3^)	401.45 (11)	1439.99 (10)	1763.5 (2)
*Z*	2	8	4
Radiation type	Cu *K*α	Cu *K*α	Cu *K*α
μ (mm^−1^)	1.07	0.86	2.12
Crystal size (mm)	0.45 × 0.30 × 0.25	0.60 × 0.45 × 0.35	0.50 × 0.08 × 0.05

Data collection			
Diffractometer	Rigaku Xcalibur, Ruby	Rigaku Xcalibur, Ruby	Rigaku Xcalibur, Ruby
Absorption correction	Multi-scan (*CrysAlis PRO*; Rigaku OD, 2018 ▸)	Multi-scan (*CrysAlis PRO*; Rigaku OD, 2018 ▸)	Multi-scan (*CrysAlis PRO*; Rigaku OD, 2018 ▸)
*T*_min_, *T*_max_	0.742, 1.000	0.720, 1.000	0.934, 1.000
No. of measured, independent and observed [*I* > 2σ(*I*)] reflections	2652, 1598, 1124	22195, 2976, 2489	6703, 3563, 2207
*R*_int_	0.024	0.070	0.052
(sin θ/λ)_max_ (Å^−1^)	0.630	0.630	0.629

Refinement			
*R*[*F*^2^ > 2σ(*F* ^2^)], *wR*(*F* ^2^), *S*	0.047, 0.145, 1.02	0.036, 0.098, 1.02	0.054, 0.151, 1.01
No. of reflections	1598	2976	3563
No. of parameters	132	242	261
No. of restraints	0	2	4
H-atom treatment	H atoms treated by a mixture of independent and constrained refinement	H atoms treated by a mixture of independent and constrained refinement	H atoms treated by a mixture of independent and constrained refinement
Δρ_max_, Δρ_min_ (e Å^−3^)	0.18, −0.17	0.17, −0.15	0.23, −0.22
Absolute structure	–	Flack *x* determined using 1053 quotients [(*I* ^+^)−(*I* ^−^)]/[(*I* ^+^)+(*I* ^−^)] (Parsons *et al.*, 2013 ▸)	–
Absolute structure parameter	–	0.2 (2)	–

Computer programs: *CrysAlis PRO* (Rigaku OD, 2018 ▸), *SHELXT2018/2* (Sheldrick, 2015*a* ▸), *SHELXL2014/7* (Sheldrick, 2015*b* ▸), *XP* in *SHELXTL* (Sheldrick, 2008 ▸) and *publCIF* (Westrip, 2010 ▸).

## supplementary crystallographic information

### 6-Nitroquinazolin-4(3H)-one (I) Crystal data

**Table:**

C_8_H_5_N_3_O_3_	*F*(000) = 196
*M**_r_* = 191.15	*D*_x_ = 1.581 Mg m^−^^3^
Triclinic, *P*1	Melting point: 560(2) K
*a* = 5.5587 (9) Å	Cu *K*α radiation, λ = 1.54184 Å
*b* = 8.6673 (13) Å	Cell parameters from 950 reflections
*c* = 8.7649 (12) Å	θ = 5.3–74.5°
α = 105.654 (12)°	µ = 1.07 mm^−^^1^
β = 98.560 (13)°	*T* = 293 K
γ = 90.784 (13)°	Prism, colourless
*V* = 401.45 (11) Å^3^	0.45 × 0.30 × 0.25 mm
*Z* = 2	

### 6-Nitroquinazolin-4(3H)-one (I) Data collection

**Table:**

Rigaku Xcalibur, Ruby diffractometer	1598 independent reflections
Radiation source: Enhance (Cu) X-ray Source	1124 reflections with *I* > 2σ(*I*)
Graphite monochromator	*R*_int_ = 0.024
Detector resolution: 10.2576 pixels mm^-1^	θ_max_ = 76.3°, θ_min_ = 5.3°
ω scans	*h* = −6→7
Absorption correction: multi-scan (CrysAlisPro; Rigaku OD, 2018)	*k* = −7→10
*T*_min_ = 0.742, *T*_max_ = 1.000	*l* = −10→9
2652 measured reflections	

### 6-Nitroquinazolin-4(3H)-one (I) Refinement

**Table:**

Refinement on *F*^2^	Hydrogen site location: mixed
Least-squares matrix: full	H atoms treated by a mixture of independent and constrained refinement
*R*[*F*^2^ > 2σ(*F*^2^)] = 0.047	*w* = 1/[σ^2^(*F*_o_^2^) + (0.0615*P*)^2^] where *P* = (*F*_o_^2^ + 2*F*_c_^2^)/3
*wR*(*F*^2^) = 0.145	(Δ/σ)_max_ < 0.001
*S* = 1.02	Δρ_max_ = 0.18 e Å^−^^3^
1598 reflections	Δρ_min_ = −0.17 e Å^−^^3^
132 parameters	Extinction correction: SHELXL2014/7 (Sheldrick, 2015b), Fc^*^=kFc[1+0.001xFc^2^λ^3^/sin(2θ)]^-1/4^
0 restraints	Extinction coefficient: 0.012 (3)
Primary atom site location: structure-invariant direct methods	

### 6-Nitroquinazolin-4(3H)-one (I) Special details

**Table:**

Geometry. All esds (except the esd in the dihedral angle between two l.s. planes) are estimated using the full covariance matrix. The cell esds are taken into account individually in the estimation of esds in distances, angles and torsion angles; correlations between esds in cell parameters are only used when they are defined by crystal symmetry. An approximate (isotropic) treatment of cell esds is used for estimating esds involving l.s. planes.

### 6-Nitroquinazolin-4(3H)-one (I) Fractional atomic coordinates and isotropic or equivalent isotropic displacement parameters (Å^2^)

**Table:**

	*x*	*y*	*z*	*U*_iso_*/*U*_eq_	
O1	0.6353 (3)	0.19086 (17)	0.52777 (19)	0.0644 (5)	
O2	0.7328 (5)	0.9420 (2)	0.8525 (3)	0.0988 (8)	
O3	0.8935 (3)	0.7763 (2)	0.6717 (2)	0.0739 (5)	
N1	0.1305 (3)	0.3183 (2)	0.8279 (2)	0.0587 (5)	
C2	0.1601 (4)	0.1736 (3)	0.7482 (3)	0.0585 (5)	
H2	0.0576	0.0929	0.7592	0.070*	
N3	0.3277 (4)	0.1287 (2)	0.6497 (2)	0.0566 (5)	
C4	0.4881 (4)	0.2343 (2)	0.6209 (2)	0.0517 (5)	
C4A	0.4645 (4)	0.4012 (2)	0.7094 (2)	0.0476 (5)	
C5	0.6197 (4)	0.5228 (2)	0.6955 (3)	0.0519 (5)	
H5	0.7399	0.4998	0.6306	0.062*	
C6	0.5897 (4)	0.6775 (2)	0.7807 (3)	0.0529 (5)	
C7	0.4120 (4)	0.7162 (2)	0.8796 (3)	0.0569 (5)	
H7	0.3956	0.8224	0.9349	0.068*	
C8	0.2626 (4)	0.5968 (2)	0.8944 (3)	0.0567 (5)	
H8	0.1446	0.6214	0.9607	0.068*	
C8A	0.2866 (4)	0.4359 (2)	0.8092 (2)	0.0507 (5)	
N9	0.7504 (4)	0.8071 (2)	0.7678 (3)	0.0635 (5)	
H3	0.334 (5)	0.038 (3)	0.598 (3)	0.057 (6)*	

### 6-Nitroquinazolin-4(3H)-one (I) Atomic displacement parameters (Å^2^)

**Table:**

	*U*^11^	*U*^22^	*U*^33^	*U*^12^	*U*^13^	*U*^23^
O1	0.0731 (10)	0.0452 (8)	0.0736 (11)	0.0019 (7)	0.0352 (9)	0.0014 (7)
O2	0.1272 (18)	0.0417 (9)	0.1230 (17)	−0.0165 (10)	0.0525 (15)	−0.0014 (9)
O3	0.0730 (11)	0.0618 (10)	0.0918 (13)	−0.0050 (8)	0.0287 (10)	0.0215 (9)
N1	0.0605 (10)	0.0490 (9)	0.0681 (11)	−0.0004 (7)	0.0260 (9)	0.0102 (8)
C2	0.0621 (12)	0.0457 (11)	0.0690 (14)	−0.0031 (9)	0.0213 (11)	0.0126 (9)
N3	0.0674 (11)	0.0368 (8)	0.0646 (11)	0.0004 (7)	0.0226 (9)	0.0058 (8)
C4	0.0553 (11)	0.0431 (10)	0.0557 (11)	0.0035 (8)	0.0169 (9)	0.0073 (8)
C4A	0.0534 (10)	0.0398 (9)	0.0486 (10)	0.0030 (8)	0.0129 (8)	0.0082 (8)
C5	0.0549 (11)	0.0478 (11)	0.0542 (11)	0.0042 (8)	0.0154 (9)	0.0122 (8)
C6	0.0571 (11)	0.0430 (10)	0.0579 (11)	−0.0013 (8)	0.0111 (9)	0.0118 (8)
C7	0.0674 (13)	0.0396 (10)	0.0600 (12)	0.0067 (9)	0.0143 (10)	0.0052 (8)
C8	0.0617 (12)	0.0464 (11)	0.0606 (12)	0.0070 (9)	0.0211 (10)	0.0061 (9)
C8A	0.0535 (11)	0.0436 (10)	0.0542 (11)	0.0027 (8)	0.0135 (9)	0.0092 (8)
N9	0.0694 (12)	0.0453 (10)	0.0744 (12)	−0.0043 (8)	0.0133 (10)	0.0138 (8)

### 6-Nitroquinazolin-4(3H)-one (I) Geometric parameters (Å, º)

**Table:**

O1—C4	1.233 (2)	C4A—C5	1.395 (3)
O2—N9	1.218 (2)	C4A—C8A	1.399 (3)
O3—N9	1.223 (2)	C5—C6	1.374 (3)
N1—C2	1.287 (3)	C5—H5	0.9300
N1—C8A	1.388 (3)	C6—C7	1.395 (3)
C2—N3	1.354 (3)	C6—N9	1.464 (3)
C2—H2	0.9300	C7—C8	1.363 (3)
N3—C4	1.366 (3)	C7—H7	0.9300
N3—H3	0.80 (3)	C8—C8A	1.412 (3)
C4—C4A	1.463 (3)	C8—H8	0.9300
			
C2—N1—C8A	115.80 (17)	C5—C6—C7	122.54 (19)
N1—C2—N3	125.57 (19)	C5—C6—N9	118.89 (18)
N1—C2—H2	117.2	C7—C6—N9	118.57 (18)
N3—C2—H2	117.2	C8—C7—C6	119.31 (18)
C2—N3—C4	123.57 (17)	C8—C7—H7	120.3
C2—N3—H3	122.0 (18)	C6—C7—H7	120.3
C4—N3—H3	114.3 (18)	C7—C8—C8A	120.21 (19)
O1—C4—N3	122.34 (17)	C7—C8—H8	119.9
O1—C4—C4A	124.23 (18)	C8A—C8—H8	119.9
N3—C4—C4A	113.43 (16)	N1—C8A—C4A	122.73 (18)
C5—C4A—C8A	120.89 (18)	N1—C8A—C8	118.18 (18)
C5—C4A—C4	120.21 (17)	C4A—C8A—C8	119.09 (19)
C8A—C4A—C4	118.90 (18)	O2—N9—O3	122.9 (2)
C6—C5—C4A	117.95 (18)	O2—N9—C6	118.0 (2)
C6—C5—H5	121.0	O3—N9—C6	119.03 (18)
C4A—C5—H5	121.0		
			
C8A—N1—C2—N3	−0.1 (4)	C6—C7—C8—C8A	−0.5 (4)
N1—C2—N3—C4	−0.7 (4)	C2—N1—C8A—C4A	0.5 (3)
C2—N3—C4—O1	−178.3 (2)	C2—N1—C8A—C8	−179.8 (2)
C2—N3—C4—C4A	1.0 (3)	C5—C4A—C8A—N1	−179.2 (2)
O1—C4—C4A—C5	−2.2 (3)	C4—C4A—C8A—N1	0.0 (3)
N3—C4—C4A—C5	178.5 (2)	C5—C4A—C8A—C8	1.1 (3)
O1—C4—C4A—C8A	178.6 (2)	C4—C4A—C8A—C8	−179.8 (2)
N3—C4—C4A—C8A	−0.7 (3)	C7—C8—C8A—N1	180.0 (2)
C8A—C4A—C5—C6	−1.0 (3)	C7—C8—C8A—C4A	−0.2 (3)
C4—C4A—C5—C6	179.8 (2)	C5—C6—N9—O2	−175.0 (2)
C4A—C5—C6—C7	0.3 (3)	C7—C6—N9—O2	4.9 (4)
C4A—C5—C6—N9	−179.9 (2)	C5—C6—N9—O3	6.0 (3)
C5—C6—C7—C8	0.5 (4)	C7—C6—N9—O3	−174.2 (2)
N9—C6—C7—C8	−179.3 (2)		

### 6-Nitroquinazolin-4(3H)-one (I) Hydrogen-bond geometry (Å, º)

**Table:**

*D*—H···*A*	*D*—H	H···*A*	*D*···*A*	*D*—H···*A*
N3—H3···O1^i^	0.80 (3)	2.02 (3)	2.814 (2)	178 (4)
C8—H8···N1^ii^	0.93	2.53	3.450 (3)	172
C2—H2···O2^iii^	0.93	2.57	3.466 (4)	163
C7—H7···O2^iv^	0.93	2.56	3.437 (3)	158

Symmetry codes: (i) −*x*+1, −*y*, −*z*+1; (ii) −*x*, −*y*+1, −*z*+2; (iii) *x*−1, *y*−1, *z*; (iv) −*x*+1, −*y*+2, −*z*+2.

### 6-Aminoquinazolin-4(3H)-one (II) Crystal data

**Table:**

C_8_H_7_N_3_O	*D*_x_ = 1.487 Mg m^−^^3^
*M**_r_* = 161.17	Melting point: 589(2) K
Orthorhombic, *P**c**a*2_1_	Cu *K*α radiation, λ = 1.54184 Å
*a* = 13.4535 (5) Å	Cell parameters from 5076 reflections
*b* = 4.9510 (2) Å	θ = 4.1–75.8°
*c* = 21.6188 (8) Å	µ = 0.86 mm^−^^1^
*V* = 1439.99 (10) Å^3^	*T* = 293 K
*Z* = 8	Prism, colourless
*F*(000) = 672	0.60 × 0.45 × 0.35 mm

### 6-Aminoquinazolin-4(3H)-one (II) Data collection

**Table:**

Rigaku Xcalibur, Ruby diffractometer	2976 independent reflections
Radiation source: Enhance (Cu) X-ray Source	2489 reflections with *I* > 2σ(*I*)
Graphite monochromator	*R*_int_ = 0.070
Detector resolution: 10.2576 pixels mm^-1^	θ_max_ = 76.1°, θ_min_ = 4.1°
ω scans	*h* = −16→16
Absorption correction: multi-scan (CrysAlisPro; Rigaku OD, 2018)	*k* = −6→6
*T*_min_ = 0.720, *T*_max_ = 1.000	*l* = −26→27
22195 measured reflections	

### 6-Aminoquinazolin-4(3H)-one (II) Refinement

**Table:**

Refinement on *F*^2^	H atoms treated by a mixture of independent and constrained refinement
Least-squares matrix: full	*w* = 1/[σ^2^(*F*_o_^2^) + (0.0516*P*)^2^ + 0.144*P*] where *P* = (*F*_o_^2^ + 2*F*_c_^2^)/3
*R*[*F*^2^ > 2σ(*F*^2^)] = 0.036	(Δ/σ)_max_ = 0.001
*wR*(*F*^2^) = 0.098	Δρ_max_ = 0.17 e Å^−^^3^
*S* = 1.02	Δρ_min_ = −0.14 e Å^−^^3^
2976 reflections	Extinction correction: SHELXL-2014/7 (Sheldrick, 2015), Fc^*^=kFc[1+0.001xFc^2^λ^3^/sin(2θ)]^-1/4^
242 parameters	Extinction coefficient: 0.0034 (4)
2 restraints	Absolute structure: Flack *x* determined using 1053 quotients [(*I*^+^)-(*I*^-^)]/[(*I*^+^)+(*I*^-^)] (Parsons *et al.*, 2013)
Primary atom site location: structure-invariant direct methods	Absolute structure parameter: 0.2 (2)
Hydrogen site location: mixed	

### 6-Aminoquinazolin-4(3H)-one (II) Special details

**Table:**

Geometry. All esds (except the esd in the dihedral angle between two l.s. planes) are estimated using the full covariance matrix. The cell esds are taken into account individually in the estimation of esds in distances, angles and torsion angles; correlations between esds in cell parameters are only used when they are defined by crystal symmetry. An approximate (isotropic) treatment of cell esds is used for estimating esds involving l.s. planes.

### 6-Aminoquinazolin-4(3H)-one (II) Fractional atomic coordinates and isotropic or equivalent isotropic displacement parameters (Å^2^)

**Table:**

	*x*	*y*	*z*	*U*_iso_*/*U*_eq_	
O1A	0.40087 (16)	0.0190 (4)	0.46170 (11)	0.0394 (5)	
N1A	0.6791 (2)	0.2101 (6)	0.39888 (13)	0.0427 (7)	
C2A	0.6607 (2)	0.0205 (7)	0.43824 (16)	0.0408 (8)	
H2A	0.7142	−0.0786	0.4532	0.049*	
N3A	0.5680 (2)	−0.0455 (6)	0.45958 (13)	0.0373 (6)	
C4A	0.4828 (2)	0.0850 (6)	0.44094 (14)	0.0337 (7)	
C4AA	0.4994 (2)	0.3010 (6)	0.39636 (14)	0.0338 (7)	
C5A	0.4197 (2)	0.4509 (6)	0.37325 (14)	0.0361 (7)	
H5A	0.3555	0.4132	0.3867	0.043*	
C6A	0.4353 (2)	0.6566 (7)	0.33025 (15)	0.0368 (7)	
C7A	0.5343 (3)	0.7079 (7)	0.31113 (15)	0.0394 (8)	
H7A	0.5464	0.8449	0.2827	0.047*	
C8A	0.6122 (3)	0.5608 (7)	0.33352 (16)	0.0412 (8)	
H8A	0.6763	0.5982	0.3198	0.049*	
C8AA	0.5971 (3)	0.3541 (6)	0.37691 (15)	0.0364 (7)	
N9A	0.3578 (3)	0.8001 (6)	0.30523 (15)	0.0469 (8)	
O1B	0.53939 (17)	0.5050 (4)	0.53859 (12)	0.0403 (6)	
N1B	0.2624 (2)	0.3084 (6)	0.60178 (14)	0.0451 (7)	
C2B	0.2799 (2)	0.4952 (7)	0.56163 (16)	0.0430 (8)	
H2B	0.2257	0.5891	0.5458	0.052*	
N3B	0.3720 (2)	0.5655 (5)	0.54065 (13)	0.0385 (6)	
C4B	0.4570 (3)	0.4384 (6)	0.55929 (14)	0.0332 (7)	
C4AB	0.4421 (2)	0.2222 (6)	0.60434 (15)	0.0332 (7)	
C5B	0.5223 (3)	0.0742 (6)	0.62695 (14)	0.0367 (7)	
H5B	0.5863	0.1136	0.6134	0.044*	
C6B	0.5075 (3)	−0.1315 (6)	0.66954 (15)	0.0371 (7)	
C7B	0.4094 (3)	−0.1838 (7)	0.68957 (15)	0.0409 (8)	
H7B	0.3983	−0.3203	0.7183	0.049*	
C8B	0.3303 (3)	−0.0383 (7)	0.66766 (15)	0.0415 (8)	
H8B	0.2666	−0.0768	0.6818	0.050*	
C8AB	0.3444 (2)	0.1679 (6)	0.62419 (15)	0.0373 (7)	
N9B	0.5875 (3)	−0.2736 (7)	0.69421 (15)	0.0470 (8)	
H3A	0.560 (2)	−0.190 (7)	0.4877 (16)	0.039 (10)*	
H3B	0.383 (3)	0.702 (7)	0.5125 (17)	0.059 (12)*	
H9AA	0.373 (3)	0.957 (9)	0.2838 (19)	0.054 (12)*	
H9BB	0.638 (3)	−0.297 (10)	0.667 (2)	0.071 (14)*	
H9BA	0.571 (3)	−0.431 (9)	0.715 (2)	0.062 (12)*	
H9AB	0.307 (3)	0.804 (8)	0.3255 (19)	0.050 (12)*	

### 6-Aminoquinazolin-4(3H)-one (II) Atomic displacement parameters (Å^2^)

**Table:**

	*U*^11^	*U*^22^	*U*^33^	*U*^12^	*U*^13^	*U*^23^
O1A	0.0381 (13)	0.0371 (12)	0.0429 (11)	−0.0020 (9)	0.0049 (10)	0.0039 (10)
N1A	0.0368 (15)	0.0409 (16)	0.0505 (18)	−0.0034 (13)	0.0035 (13)	0.0010 (13)
C2A	0.0340 (18)	0.0394 (19)	0.0491 (19)	0.0022 (14)	0.0003 (15)	0.0011 (16)
N3A	0.0399 (15)	0.0334 (14)	0.0385 (15)	−0.0004 (11)	0.0010 (13)	0.0035 (13)
C4A	0.0358 (17)	0.0317 (15)	0.0337 (16)	−0.0019 (12)	0.0017 (13)	−0.0064 (13)
C4AA	0.0376 (17)	0.0307 (15)	0.0332 (16)	−0.0035 (13)	0.0013 (12)	−0.0058 (12)
C5A	0.0375 (17)	0.0344 (16)	0.0363 (17)	−0.0031 (13)	0.0016 (14)	−0.0022 (14)
C6A	0.0446 (19)	0.0337 (16)	0.0322 (16)	0.0004 (14)	0.0007 (14)	−0.0045 (13)
C7A	0.053 (2)	0.0329 (17)	0.0320 (18)	−0.0053 (14)	0.0051 (14)	0.0019 (14)
C8A	0.043 (2)	0.0395 (18)	0.0409 (18)	−0.0083 (14)	0.0084 (15)	−0.0016 (14)
C8AA	0.0383 (17)	0.0338 (17)	0.0371 (17)	−0.0015 (14)	0.0043 (14)	−0.0029 (14)
N9A	0.049 (2)	0.0437 (17)	0.0482 (18)	0.0016 (14)	−0.0013 (15)	0.0106 (15)
O1B	0.0384 (13)	0.0388 (12)	0.0437 (12)	−0.0036 (10)	0.0049 (11)	0.0058 (10)
N1B	0.0365 (15)	0.0463 (17)	0.0525 (17)	−0.0017 (12)	0.0019 (13)	0.0057 (13)
C2B	0.0386 (19)	0.0428 (18)	0.0475 (19)	0.0008 (14)	−0.0012 (15)	0.0015 (16)
N3B	0.0422 (16)	0.0333 (15)	0.0399 (15)	−0.0017 (11)	0.0032 (13)	0.0033 (12)
C4B	0.0393 (19)	0.0291 (16)	0.0313 (16)	−0.0029 (12)	0.0016 (13)	−0.0014 (13)
C4AB	0.0384 (18)	0.0299 (15)	0.0314 (16)	−0.0033 (13)	0.0040 (13)	−0.0031 (13)
C5B	0.0389 (19)	0.0350 (16)	0.0361 (17)	−0.0041 (14)	0.0047 (14)	−0.0041 (13)
C6B	0.0457 (19)	0.0323 (16)	0.0332 (17)	−0.0005 (14)	−0.0009 (14)	−0.0015 (14)
C7B	0.051 (2)	0.0362 (18)	0.0350 (17)	−0.0071 (15)	0.0032 (15)	0.0027 (14)
C8B	0.0396 (19)	0.0450 (19)	0.0399 (18)	−0.0070 (14)	0.0101 (14)	−0.0022 (15)
C8AB	0.0391 (17)	0.0335 (16)	0.0392 (17)	−0.0032 (13)	0.0018 (14)	−0.0020 (14)
N9B	0.0504 (19)	0.0457 (18)	0.0448 (18)	0.0022 (14)	0.0025 (15)	0.0079 (14)

### 6-Aminoquinazolin-4(3H)-one (II) Geometric parameters (Å, º)

**Table:**

O1A—C4A	1.235 (4)	O1B—C4B	1.239 (4)
N1A—C2A	1.291 (5)	N1B—C2B	1.290 (5)
N1A—C8AA	1.397 (4)	N1B—C8AB	1.391 (4)
C2A—N3A	1.369 (4)	C2B—N3B	1.364 (4)
C2A—H2A	0.9300	C2B—H2B	0.9300
N3A—C4A	1.376 (4)	N3B—C4B	1.366 (4)
N3A—H3A	0.94 (3)	N3B—H3B	0.92 (2)
C4A—C4AA	1.457 (4)	C4B—C4AB	1.461 (5)
C4AA—C5A	1.397 (4)	C4AB—C5B	1.393 (5)
C4AA—C8AA	1.405 (4)	C4AB—C8AB	1.408 (4)
C5A—C6A	1.395 (5)	C5B—C6B	1.387 (5)
C5A—H5A	0.9300	C5B—H5B	0.9300
C6A—N9A	1.374 (4)	C6B—N9B	1.392 (5)
C6A—C7A	1.417 (5)	C6B—C7B	1.413 (5)
C7A—C8A	1.365 (5)	C7B—C8B	1.370 (5)
C7A—H7A	0.9300	C7B—H7B	0.9300
C8A—C8AA	1.403 (5)	C8B—C8AB	1.400 (5)
C8A—H8A	0.9300	C8B—H8B	0.9300
N9A—H9AA	0.93 (4)	N9B—H9BB	0.90 (5)
N9A—H9AB	0.81 (4)	N9B—H9BA	0.93 (5)
			
C2A—N1A—C8AA	116.3 (3)	C2B—N1B—C8AB	116.6 (3)
N1A—C2A—N3A	124.8 (3)	N1B—C2B—N3B	124.9 (3)
N1A—C2A—H2A	117.6	N1B—C2B—H2B	117.6
N3A—C2A—H2A	117.6	N3B—C2B—H2B	117.6
C2A—N3A—C4A	123.2 (3)	C2B—N3B—C4B	123.0 (3)
C2A—N3A—H3A	120 (2)	C2B—N3B—H3B	123 (2)
C4A—N3A—H3A	117 (2)	C4B—N3B—H3B	114 (2)
O1A—C4A—N3A	120.9 (3)	O1B—C4B—N3B	121.3 (3)
O1A—C4A—C4AA	124.9 (3)	O1B—C4B—C4AB	123.9 (3)
N3A—C4A—C4AA	114.3 (3)	N3B—C4B—C4AB	114.7 (3)
C5A—C4AA—C8AA	120.8 (3)	C5B—C4AB—C8AB	121.0 (3)
C5A—C4AA—C4A	120.6 (3)	C5B—C4AB—C4B	120.9 (3)
C8AA—C4AA—C4A	118.6 (3)	C8AB—C4AB—C4B	118.1 (3)
C6A—C5A—C4AA	120.7 (3)	C6B—C5B—C4AB	120.6 (3)
C6A—C5A—H5A	119.6	C6B—C5B—H5B	119.7
C4AA—C5A—H5A	119.6	C4AB—C5B—H5B	119.7
N9A—C6A—C5A	121.7 (3)	C5B—C6B—N9B	121.0 (3)
N9A—C6A—C7A	120.4 (3)	C5B—C6B—C7B	118.1 (3)
C5A—C6A—C7A	117.8 (3)	N9B—C6B—C7B	120.8 (3)
C8A—C7A—C6A	121.5 (3)	C8B—C7B—C6B	121.6 (3)
C8A—C7A—H7A	119.3	C8B—C7B—H7B	119.2
C6A—C7A—H7A	119.3	C6B—C7B—H7B	119.2
C7A—C8A—C8AA	121.0 (3)	C7B—C8B—C8AB	120.7 (3)
C7A—C8A—H8A	119.5	C7B—C8B—H8B	119.7
C8AA—C8A—H8A	119.5	C8AB—C8B—H8B	119.7
N1A—C8AA—C8A	119.0 (3)	N1B—C8AB—C8B	119.4 (3)
N1A—C8AA—C4AA	122.8 (3)	N1B—C8AB—C4AB	122.6 (3)
C8A—C8AA—C4AA	118.2 (3)	C8B—C8AB—C4AB	118.0 (3)
C6A—N9A—H9AA	117 (3)	C6B—N9B—H9BB	113 (3)
C6A—N9A—H9AB	116 (3)	C6B—N9B—H9BA	116 (3)
H9AA—N9A—H9AB	116 (4)	H9BB—N9B—H9BA	113 (4)
			
C8AA—N1A—C2A—N3A	−0.4 (5)	C8AB—N1B—C2B—N3B	−0.9 (5)
N1A—C2A—N3A—C4A	0.2 (5)	N1B—C2B—N3B—C4B	1.6 (5)
C2A—N3A—C4A—O1A	−179.7 (3)	C2B—N3B—C4B—O1B	178.9 (3)
C2A—N3A—C4A—C4AA	−0.1 (4)	C2B—N3B—C4B—C4AB	−0.8 (4)
O1A—C4A—C4AA—C5A	−0.5 (5)	O1B—C4B—C4AB—C5B	−0.2 (5)
N3A—C4A—C4AA—C5A	179.9 (3)	N3B—C4B—C4AB—C5B	179.5 (3)
O1A—C4A—C4AA—C8AA	179.8 (3)	O1B—C4B—C4AB—C8AB	179.8 (3)
N3A—C4A—C4AA—C8AA	0.2 (4)	N3B—C4B—C4AB—C8AB	−0.5 (4)
C8AA—C4AA—C5A—C6A	0.1 (5)	C8AB—C4AB—C5B—C6B	0.2 (5)
C4A—C4AA—C5A—C6A	−179.6 (3)	C4B—C4AB—C5B—C6B	−179.8 (3)
C4AA—C5A—C6A—N9A	177.6 (3)	C4AB—C5B—C6B—N9B	−177.5 (3)
C4AA—C5A—C6A—C7A	−0.1 (5)	C4AB—C5B—C6B—C7B	−0.6 (5)
N9A—C6A—C7A—C8A	−177.4 (3)	C5B—C6B—C7B—C8B	0.4 (5)
C5A—C6A—C7A—C8A	0.4 (5)	N9B—C6B—C7B—C8B	177.3 (3)
C6A—C7A—C8A—C8AA	−0.6 (5)	C6B—C7B—C8B—C8AB	0.2 (5)
C2A—N1A—C8AA—C8A	−179.3 (3)	C2B—N1B—C8AB—C8B	−179.7 (3)
C2A—N1A—C8AA—C4AA	0.6 (5)	C2B—N1B—C8AB—C4AB	−0.5 (5)
C7A—C8A—C8AA—N1A	−179.6 (3)	C7B—C8B—C8AB—N1B	178.6 (3)
C7A—C8A—C8AA—C4AA	0.5 (5)	C7B—C8B—C8AB—C4AB	−0.6 (5)
C5A—C4AA—C8AA—N1A	179.8 (3)	C5B—C4AB—C8AB—N1B	−178.8 (3)
C4A—C4AA—C8AA—N1A	−0.5 (4)	C4B—C4AB—C8AB—N1B	1.2 (5)
C5A—C4AA—C8AA—C8A	−0.3 (4)	C5B—C4AB—C8AB—C8B	0.4 (4)
C4A—C4AA—C8AA—C8A	179.4 (3)	C4B—C4AB—C8AB—C8B	−179.6 (3)

### 6-Aminoquinazolin-4(3H)-one (II) Hydrogen-bond geometry (Å, º)

**Table:**

*D*—H···*A*	*D*—H	H···*A*	*D*···*A*	*D*—H···*A*
N9*A*—H9*AA*···N9*B*^i^	0.93 (4)	2.55 (4)	3.435 (5)	160 (4)
N9*A*—H9*AB*···N1*A*^ii^	0.81 (4)	2.34 (4)	3.144 (5)	170 (4)
N9*B*—H9*BB*···N1*B*^iii^	0.91 (4)	2.19 (4)	3.092 (5)	174 (4)
N3*A*—H3*A*···O1*B*^iv^	0.95 (3)	1.89 (3)	2.832 (4)	175 (3)
N3*B*—H3*B*···O1*A*^v^	0.92 (4)	1.93 (4)	2.847 (3)	173 (4)

Symmetry codes: (i) −*x*+1, −*y*+1, *z*−1/2; (ii) *x*−1/2, −*y*+1, *z*; (iii) *x*+1/2, −*y*, *z*; (iv) *x*, *y*−1, *z*; (v) *x*, *y*+1, *z*.

### 4-Aminoquinazolin-1-ium chloride–4-aminoquinazoline–water (1/1/2) (III) Crystal data

**Table:**

C_8_H_8_N_3_^+^·Cl^−^·C_8_H_7_N_3_·2H_2_O	*F*(000) = 760
*M**_r_* = 362.82	*D*_x_ = 1.367 Mg m^−^^3^
Monoclinic, *P*2_1_/*n*	Cu *K*α radiation, λ = 1.54184 Å
*a* = 14.3512 (12) Å	Cell parameters from 1071 reflections
*b* = 7.5867 (6) Å	θ = 4.0–71.2°
*c* = 16.2282 (9) Å	µ = 2.12 mm^−^^1^
β = 93.544 (7)°	*T* = 298 K
*V* = 1763.5 (2) Å^3^	Needle, colourless
*Z* = 4	0.50 × 0.08 × 0.05 mm

### 4-Aminoquinazolin-1-ium chloride–4-aminoquinazoline–water (1/1/2) (III) Data collection

**Table:**

Rigaku Xcalibur, Ruby diffractometer	3563 independent reflections
Radiation source: Enhance (Cu) X-ray Source	2207 reflections with *I* > 2σ(*I*)
Graphite monochromator	*R*_int_ = 0.052
Detector resolution: 10.2576 pixels mm^-1^	θ_max_ = 75.8°, θ_min_ = 4.0°
ω scans	*h* = −17→15
Absorption correction: multi-scan (CrysAlisPro; Rigaku OD, 2018)	*k* = −9→9
*T*_min_ = 0.934, *T*_max_ = 1.000	*l* = −15→19
6703 measured reflections	

### 4-Aminoquinazolin-1-ium chloride–4-aminoquinazoline–water (1/1/2) (III) Refinement

**Table:**

Refinement on *F*^2^	Primary atom site location: structure-invariant direct methods
Least-squares matrix: full	Hydrogen site location: mixed
*R*[*F*^2^ > 2σ(*F*^2^)] = 0.054	H atoms treated by a mixture of independent and constrained refinement
*wR*(*F*^2^) = 0.151	*w* = 1/[σ^2^(*F*_o_^2^) + (0.0514*P*)^2^] where *P* = (*F*_o_^2^ + 2*F*_c_^2^)/3
*S* = 1.00	(Δ/σ)_max_ = 0.003
3563 reflections	Δρ_max_ = 0.23 e Å^−^^3^
261 parameters	Δρ_min_ = −0.21 e Å^−^^3^
4 restraints	

### 4-Aminoquinazolin-1-ium chloride–4-aminoquinazoline–water (1/1/2) (III) Special details

**Table:**

Geometry. All esds (except the esd in the dihedral angle between two l.s. planes) are estimated using the full covariance matrix. The cell esds are taken into account individually in the estimation of esds in distances, angles and torsion angles; correlations between esds in cell parameters are only used when they are defined by crystal symmetry. An approximate (isotropic) treatment of cell esds is used for estimating esds involving l.s. planes.

### 4-Aminoquinazolin-1-ium chloride–4-aminoquinazoline–water (1/1/2) (III) Fractional atomic coordinates and isotropic or equivalent isotropic displacement parameters (Å^2^)

**Table:**

	*x*	*y*	*z*	*U*_iso_*/*U*_eq_	
Cl1	0.38300 (8)	0.88404 (15)	0.76044 (5)	0.0719 (3)	
O1W	0.3293 (2)	0.4885 (5)	0.72013 (16)	0.0732 (8)	
O2W	0.4789 (2)	0.2659 (6)	0.7806 (2)	0.0896 (10)	
N1A	0.28852 (17)	0.9176 (4)	0.34296 (12)	0.0423 (6)	
C2A	0.3708 (2)	0.8430 (4)	0.35562 (16)	0.0440 (7)	
H2AA	0.4007	0.8052	0.3095	0.053*	
N3A	0.41460 (17)	0.8174 (4)	0.42915 (13)	0.0408 (5)	
C4A	0.3713 (2)	0.8717 (4)	0.49712 (16)	0.0396 (6)	
C4AA	0.28014 (19)	0.9559 (4)	0.48907 (16)	0.0389 (6)	
C5A	0.2313 (2)	1.0135 (5)	0.55595 (17)	0.0484 (7)	
H5AA	0.2578	1.0010	0.6094	0.058*	
C6A	0.1456 (2)	1.0874 (5)	0.5433 (2)	0.0551 (8)	
H6AA	0.1133	1.1248	0.5881	0.066*	
C7A	0.1056 (2)	1.1074 (5)	0.4628 (2)	0.0527 (7)	
H7AA	0.0468	1.1584	0.4546	0.063*	
C8A	0.1520 (2)	1.0528 (4)	0.39587 (17)	0.0469 (7)	
H8AA	0.1253	1.0664	0.3426	0.056*	
C8AA	0.23995 (19)	0.9765 (4)	0.40943 (16)	0.0385 (6)	
N9A	0.41535 (19)	0.8422 (4)	0.56770 (14)	0.0505 (7)	
N1B	0.28423 (18)	0.4265 (4)	0.32016 (12)	0.0435 (6)	
C2B	0.3657 (2)	0.3578 (4)	0.34201 (16)	0.0447 (7)	
H2BA	0.4010	0.3203	0.2991	0.054*	
N3B	0.40470 (17)	0.3348 (4)	0.41844 (14)	0.0431 (6)	
C4B	0.35638 (19)	0.3916 (4)	0.48186 (15)	0.0382 (6)	
C4AB	0.26521 (19)	0.4695 (4)	0.46662 (15)	0.0359 (5)	
C5B	0.2086 (2)	0.5269 (4)	0.52924 (16)	0.0426 (6)	
H5BA	0.2304	0.5181	0.5843	0.051*	
C6B	0.1221 (2)	0.5954 (4)	0.50991 (19)	0.0486 (7)	
H6BA	0.0856	0.6345	0.5516	0.058*	
C7B	0.0883 (2)	0.6069 (4)	0.42737 (19)	0.0481 (7)	
H7BA	0.0290	0.6522	0.4146	0.058*	
C8B	0.1419 (2)	0.5522 (4)	0.36512 (16)	0.0439 (7)	
H8BA	0.1189	0.5615	0.3104	0.053*	
C8AB	0.23112 (19)	0.4821 (4)	0.38354 (15)	0.0383 (6)	
N9B	0.39542 (19)	0.3709 (4)	0.55724 (14)	0.0493 (7)	
H1A	0.259 (3)	0.929 (5)	0.284 (2)	0.060 (10)*	
H9AA	0.472 (3)	0.789 (5)	0.569 (2)	0.062 (11)*	
H9AB	0.390 (4)	0.880 (7)	0.617 (3)	0.103 (17)*	
H9BA	0.453 (3)	0.308 (5)	0.565 (2)	0.057 (10)*	
H9BB	0.370 (3)	0.406 (5)	0.596 (2)	0.047 (9)*	
H1W1	0.345 (4)	0.601 (4)	0.733 (3)	0.11 (2)*	
H2W1	0.271 (2)	0.497 (11)	0.736 (4)	0.19 (4)*	
H1W2	0.437 (5)	0.346 (8)	0.759 (5)	0.190*	
H2W2	0.437 (4)	0.183 (7)	0.766 (4)	0.15 (3)*	

### 4-Aminoquinazolin-1-ium chloride–4-aminoquinazoline–water (1/1/2) (III) Atomic displacement parameters (Å^2^)

**Table:**

	*U*^11^	*U*^22^	*U*^33^	*U*^12^	*U*^13^	*U*^23^
Cl1	0.0881 (7)	0.0841 (7)	0.0431 (4)	−0.0031 (6)	0.0015 (4)	−0.0039 (4)
O1W	0.082 (2)	0.081 (2)	0.0566 (13)	0.0059 (17)	0.0021 (13)	−0.0063 (14)
O2W	0.0612 (18)	0.097 (3)	0.109 (2)	0.0033 (19)	−0.0051 (17)	−0.011 (2)
N1A	0.0404 (13)	0.0530 (16)	0.0329 (10)	−0.0007 (11)	−0.0021 (9)	0.0007 (10)
C2A	0.0400 (15)	0.0517 (18)	0.0409 (12)	−0.0035 (13)	0.0075 (11)	−0.0031 (12)
N3A	0.0333 (11)	0.0498 (15)	0.0396 (10)	0.0055 (10)	0.0051 (9)	0.0006 (10)
C4A	0.0392 (14)	0.0415 (15)	0.0382 (12)	−0.0001 (12)	0.0038 (10)	−0.0021 (11)
C4AA	0.0346 (14)	0.0380 (15)	0.0439 (13)	0.0001 (12)	0.0013 (10)	0.0021 (11)
C5A	0.0524 (18)	0.0528 (19)	0.0399 (13)	0.0034 (15)	0.0030 (12)	0.0010 (12)
C6A	0.0546 (19)	0.055 (2)	0.0569 (16)	0.0085 (16)	0.0165 (14)	−0.0047 (15)
C7A	0.0345 (15)	0.0489 (18)	0.0751 (19)	0.0105 (14)	0.0058 (13)	0.0000 (16)
C8A	0.0469 (17)	0.0473 (18)	0.0453 (13)	−0.0030 (14)	−0.0075 (12)	0.0040 (12)
C8AA	0.0377 (14)	0.0373 (14)	0.0409 (12)	−0.0032 (12)	0.0068 (10)	0.0018 (11)
N9A	0.0406 (14)	0.072 (2)	0.0390 (11)	0.0133 (13)	0.0009 (10)	−0.0045 (11)
N1B	0.0443 (13)	0.0537 (16)	0.0320 (9)	0.0025 (12)	−0.0013 (9)	−0.0011 (10)
C2B	0.0423 (15)	0.0527 (18)	0.0397 (12)	0.0029 (13)	0.0060 (11)	−0.0032 (12)
N3B	0.0338 (12)	0.0563 (16)	0.0391 (10)	0.0066 (11)	0.0010 (9)	−0.0042 (10)
C4B	0.0340 (13)	0.0436 (15)	0.0368 (11)	−0.0008 (12)	0.0008 (10)	−0.0041 (11)
C4AB	0.0337 (13)	0.0346 (14)	0.0392 (12)	−0.0010 (11)	0.0011 (10)	0.0001 (10)
C5B	0.0418 (15)	0.0499 (17)	0.0367 (12)	0.0016 (13)	0.0065 (10)	0.0000 (12)
C6B	0.0420 (16)	0.0505 (18)	0.0546 (15)	0.0029 (14)	0.0138 (12)	−0.0033 (14)
C7B	0.0314 (14)	0.0487 (18)	0.0643 (16)	0.0060 (13)	0.0031 (12)	0.0073 (15)
C8B	0.0398 (15)	0.0471 (17)	0.0441 (13)	0.0003 (13)	−0.0042 (11)	0.0064 (12)
C8AB	0.0360 (14)	0.0402 (15)	0.0388 (12)	−0.0019 (12)	0.0028 (10)	−0.0012 (11)
N9B	0.0391 (14)	0.072 (2)	0.0366 (11)	0.0112 (13)	−0.0015 (10)	−0.0058 (12)

### 4-Aminoquinazolin-1-ium chloride–4-aminoquinazoline–water (1/1/2) (III) Geometric parameters (Å, º)

**Table:**

O1W—H1W1	0.91 (2)	N9A—H9AA	0.91 (4)
O1W—H2W1	0.89 (2)	N9A—H9AB	0.94 (5)
O2W—H1W2	0.91 (2)	N1B—C2B	1.309 (4)
O2W—H2W2	0.89 (2)	N1B—C8AB	1.383 (4)
N1A—C2A	1.315 (4)	C2B—N3B	1.340 (4)
N1A—C8AA	1.393 (4)	C2B—H2BA	0.9300
N1A—H1A	1.03 (4)	N3B—C4B	1.347 (4)
C2A—N3A	1.328 (4)	C4B—N9B	1.323 (4)
C2A—H2AA	0.9300	C4B—C4AB	1.443 (4)
N3A—C4A	1.363 (4)	C4AB—C5B	1.408 (4)
C4A—N9A	1.293 (4)	C4AB—C8AB	1.409 (4)
C4A—C4AA	1.455 (4)	C5B—C6B	1.364 (4)
C4AA—C8AA	1.391 (4)	C5B—H5BA	0.9300
C4AA—C5A	1.397 (4)	C6B—C7B	1.399 (4)
C5A—C6A	1.356 (5)	C6B—H6BA	0.9300
C5A—H5AA	0.9300	C7B—C8B	1.371 (4)
C6A—C7A	1.403 (5)	C7B—H7BA	0.9300
C6A—H6AA	0.9300	C8B—C8AB	1.402 (4)
C7A—C8A	1.372 (5)	C8B—H8BA	0.9300
C7A—H7AA	0.9300	N9B—H9BA	0.95 (4)
C8A—C8AA	1.394 (4)	N9B—H9BB	0.80 (4)
C8A—H8AA	0.9300		
			
H1W1—O1W—H2W1	95 (6)	C4A—N9A—H9AB	120 (3)
H1W2—O2W—H2W2	87 (6)	H9AA—N9A—H9AB	120 (4)
C2A—N1A—C8AA	120.3 (2)	C2B—N1B—C8AB	116.4 (2)
C2A—N1A—H1A	119 (2)	N1B—C2B—N3B	128.1 (3)
C8AA—N1A—H1A	120 (2)	N1B—C2B—H2BA	115.9
N1A—C2A—N3A	125.0 (3)	N3B—C2B—H2BA	115.9
N1A—C2A—H2AA	117.5	C2B—N3B—C4B	117.4 (2)
N3A—C2A—H2AA	117.5	N9B—C4B—N3B	117.4 (3)
C2A—N3A—C4A	118.0 (2)	N9B—C4B—C4AB	122.3 (3)
N9A—C4A—N3A	116.2 (3)	N3B—C4B—C4AB	120.3 (2)
N9A—C4A—C4AA	122.9 (3)	C5B—C4AB—C8AB	119.2 (3)
N3A—C4A—C4AA	120.8 (2)	C5B—C4AB—C4B	124.1 (2)
C8AA—C4AA—C5A	119.2 (3)	C8AB—C4AB—C4B	116.7 (2)
C8AA—C4AA—C4A	116.8 (2)	C6B—C5B—C4AB	120.6 (2)
C5A—C4AA—C4A	124.0 (2)	C6B—C5B—H5BA	119.7
C6A—C5A—C4AA	120.4 (3)	C4AB—C5B—H5BA	119.7
C6A—C5A—H5AA	119.8	C5B—C6B—C7B	120.1 (3)
C4AA—C5A—H5AA	119.8	C5B—C6B—H6BA	120.0
C5A—C6A—C7A	120.0 (3)	C7B—C6B—H6BA	120.0
C5A—C6A—H6AA	120.0	C8B—C7B—C6B	120.6 (3)
C7A—C6A—H6AA	120.0	C8B—C7B—H7BA	119.7
C8A—C7A—C6A	120.9 (3)	C6B—C7B—H7BA	119.7
C8A—C7A—H7AA	119.5	C7B—C8B—C8AB	120.3 (2)
C6A—C7A—H7AA	119.5	C7B—C8B—H8BA	119.9
C7A—C8A—C8AA	118.6 (3)	C8AB—C8B—H8BA	119.9
C7A—C8A—H8AA	120.7	N1B—C8AB—C8B	119.7 (2)
C8AA—C8A—H8AA	120.7	N1B—C8AB—C4AB	121.1 (3)
C4AA—C8AA—N1A	119.0 (3)	C8B—C8AB—C4AB	119.2 (3)
C4AA—C8AA—C8A	120.8 (3)	C4B—N9B—H9BA	119 (2)
N1A—C8AA—C8A	120.2 (2)	C4B—N9B—H9BB	120 (3)
C4A—N9A—H9AA	120 (2)	H9BA—N9B—H9BB	120 (3)
			
C8AA—N1A—C2A—N3A	−0.2 (5)	C8AB—N1B—C2B—N3B	−0.2 (5)
N1A—C2A—N3A—C4A	0.2 (5)	N1B—C2B—N3B—C4B	−1.5 (5)
C2A—N3A—C4A—N9A	179.0 (3)	C2B—N3B—C4B—N9B	−179.1 (3)
C2A—N3A—C4A—C4AA	−0.3 (4)	C2B—N3B—C4B—C4AB	1.6 (5)
N9A—C4A—C4AA—C8AA	−178.8 (3)	N9B—C4B—C4AB—C5B	−1.5 (5)
N3A—C4A—C4AA—C8AA	0.4 (4)	N3B—C4B—C4AB—C5B	177.8 (3)
N9A—C4A—C4AA—C5A	0.2 (5)	N9B—C4B—C4AB—C8AB	−179.3 (3)
N3A—C4A—C4AA—C5A	179.4 (3)	N3B—C4B—C4AB—C8AB	0.0 (4)
C8AA—C4AA—C5A—C6A	0.4 (5)	C8AB—C4AB—C5B—C6B	−0.6 (5)
C4A—C4AA—C5A—C6A	−178.5 (3)	C4B—C4AB—C5B—C6B	−178.4 (3)
C4AA—C5A—C6A—C7A	−0.4 (6)	C4AB—C5B—C6B—C7B	0.9 (5)
C5A—C6A—C7A—C8A	0.1 (6)	C5B—C6B—C7B—C8B	−0.9 (5)
C6A—C7A—C8A—C8AA	0.0 (5)	C6B—C7B—C8B—C8AB	0.6 (5)
C5A—C4AA—C8AA—N1A	−179.5 (3)	C2B—N1B—C8AB—C8B	−178.1 (3)
C4A—C4AA—C8AA—N1A	−0.4 (4)	C2B—N1B—C8AB—C4AB	1.9 (4)
C5A—C4AA—C8AA—C8A	−0.2 (5)	C7B—C8B—C8AB—N1B	179.7 (3)
C4A—C4AA—C8AA—C8A	178.8 (3)	C7B—C8B—C8AB—C4AB	−0.3 (5)
C2A—N1A—C8AA—C4AA	0.3 (4)	C5B—C4AB—C8AB—N1B	−179.7 (3)
C2A—N1A—C8AA—C8A	−178.9 (3)	C4B—C4AB—C8AB—N1B	−1.8 (4)
C7A—C8A—C8AA—C4AA	0.0 (5)	C5B—C4AB—C8AB—C8B	0.3 (4)
C7A—C8A—C8AA—N1A	179.2 (3)	C4B—C4AB—C8AB—C8B	178.2 (3)

### 4-Aminoquinazolin-1-ium chloride–4-aminoquinazoline–water (1/1/2) (III) Hydrogen-bond geometry (Å, º)

**Table:**

*D*—H···*A*	*D*—H	H···*A*	*D*···*A*	*D*—H···*A*
N1*A*—H1*A*···N1*B*^i^	1.03 (3)	1.76 (3)	2.786 (3)	173 (3)
N9*A*—H9*AA*···N3*B*^ii^	0.91 (4)	2.00 (4)	2.907 (4)	175 (3)
N9*A*—H9*AB*···Cl1	0.94 (5)	2.34 (5)	3.206 (2)	153 (5)
N9*B*—H9*BA*···N3*A*^ii^	0.96 (4)	2.12 (4)	3.074 (4)	174 (3)
N9*B*—H9*BB*···O1*W*	0.79 (4)	2.22 (3)	2.999 (4)	167 (4)
O1*W*—H1*W*1···Cl1	0.90 (3)	2.25 (3)	3.157 (4)	178 (6)
O1*W*—H2*W*1···Cl1^iii^	0.89 (4)	2.37 (4)	3.183 (3)	151 (7)
O2*W*—H1*W*2···O1*W*	0.91 (7)	1.96 (7)	2.857 (5)	169 (6)
O2*W*—H2*W*2···Cl1^iv^	0.89 (5)	2.40 (5)	3.215 (4)	153 (5)

Symmetry codes: (i) −*x*+1/2, *y*+1/2, −*z*+1/2; (ii) −*x*+1, −*y*+1, −*z*+1; (iii) −*x*+1/2, *y*−1/2, −*z*+3/2; (iv) *x*, *y*−1, *z*.
